# Progress Toward Poliomyelitis Eradication — Pakistan, January 2018–September 2019

**DOI:** 10.15585/mmwr.mm6845a5

**Published:** 2019-11-15

**Authors:** Christopher H. Hsu, Milhia Kader, Abdirahman Mahamud, Kelley Bullard, Jaume Jorba, John Agbor, Malik Muhammad Safi, Hamid S. Jafari, Derek Ehrhardt

**Affiliations:** ^1^Global Immunization Division, Center for Global Health, CDC; ^2^World Health Organization, Islamabad, Pakistan; ^3^Division of Viral Diseases, National Center for Immunization and Respiratory Diseases, CDC; ^4^United Nations Children's Fund, Islamabad, Pakistan; ^5^National Emergency Operation Center, Islamabad, Pakistan; ^6^World Health Organization, Amman, Jordan.

Afghanistan and Pakistan are the only countries that continue to confirm ongoing wild poliovirus type 1 (WPV1) transmission (*1*). During January 2018–September 2019 the number of WPV1 cases in Pakistan increased, compared with the number during the previous 4 years. This report updates previous reports on Pakistan’s polio eradication activities, progress, and challenges (*2*,*3*). In 2018, Pakistan reported 12 WPV1 cases, a 50% increase from eight cases in 2017, and a 31% increase in the proportion of WPV1-positive sites under environmental surveillance (i.e., sampling of sewage to detect poliovirus). As of November 7, 2019, 80 WPV1 cases had been reported, compared with eight cases by the same time in 2018. An intensive schedule of supplementary immunization activities (SIAs)* implemented by community health workers in the core reservoirs (i.e., Karachi, Peshawar, and Quetta) where WPV1 circulation has never been interrupted, and by mobile teams, has failed to interrupt WPV1 transmission in core reservoirs and prevent WPV1 resurgence in nonreservoir areas. Sewage samples have indicated wide WPV1 transmission in nonreservoir areas in other districts and provinces. Vaccine refusals, chronically missed children, community campaign fatigue, and poor vaccination management and implementation have exacerbated the situation. To overcome challenges to vaccinating children who are chronically missed in SIAs and to attain country and global polio eradication goals, substantial changes are needed in Pakistan’s polio eradication program, including continuing cross-border coordination with Afghanistan, gaining community trust, conducting high-quality vaccination campaigns, improving oversight of field activities, and improving managerial processes to unify eradication efforts.

## Immunization Activities

**Routine immunization.** The World Health Organization (WHO) and the United Nations Children’s Fund estimated national coverage with 3 doses of oral poliovirus vaccine (OPV) received through the routine immunization program by age 1 year in Pakistan to be 75% each year during 2016–2018 (*4*). Reported 3-dose (OPV3) administrative coverage (calculated by dividing the number of doses administered by the estimated target population) is highly variable among provinces; the highest reported administrative coverage rates in 2018 were in Azad Jammu and Kashmir province (95%) and Islamabad Capital Territory (91%); the lowest were in Khyber Pakhtunkhwa (68%) and Balochistan (35%) provinces. Variation in coverage among districts is similarly high.

History of doses of OPV received (according to vaccination cards and parental recall) by children aged 6–23 months with acute flaccid paralysis (AFP) who tested negative for poliovirus (nonpolio AFP [NPAFP]^†^) is a surrogate estimate of OPV coverage in the population, with particular focus on the proportion of children who have never received OPV during SIAs or through routine immunization services (zero-dose children). Provinces and areas with the highest proportion of zero-dose children in 2018 were Gilgit-Baltistan (2.7%), Islamabad (1.2%), and Balochistan (0.9%).

**Supplementary immunization activities.** During January 2018–September 2019, seven national SIAs and nine subnational SIAs were conducted using bivalent OPV (bOPV), which contains polio vaccine virus types 1 and 3. Small-scale SIAs were implemented in response to isolation of WPV1 from environmental surveillance or from persons with AFP, using bOPV and monovalent (type 1) OPV. SIA quality was assessed in subdistricts (union councils) by intracampaign monitoring surveys and lot quality assurance sample surveys.^§^ Both methods have indicated a decline in SIA quality during 2018–2019, compared with those in previous years, with substantial numbers of children missed in union councils (up to 20% missed in Punjab and up to 17% missed in Sindh). SIA rounds using a single dose of injectable inactivated poliovirus vaccine were implemented serially in high-risk districts of Balochistan, Gilgit-Baltistan, Khyber Pakhtunkhwa, and Sindh.

**Community-based vaccination and permanent transit points.** Locally recruited community health workers in districts of core reservoirs (i.e., areas where WPV1 circulation has never been interrupted) are responsible for increasing vaccine coverage within their communities during and between SIAs through engagement with local leaders and community members. As of August 2019, a total of 19,274 community health workers had been deployed in 15 districts in Balochistan, Khyber Pakhtunkhwa, and Sindh; 85% are women, who, because of cultural and religious customs, can more easily enter homes in these areas. To identify and vaccinate children in mobile populations at high risk, 1,106 permanent transit posts (i.e., small vaccination clinics) were placed at the official border crossings with Afghanistan, along major domestic migration routes, and at railroad and bus transport hubs in all provinces.

## Surveillance Activities

**AFP surveillance**. In 2018, all provinces exceeded the target NPAFP rate of 2 per 100,000 population aged <15 years (sufficiently sensitive surveillance to detect a case of polio) and the 80% target proportion of AFP cases with collection of adequate stool specimens^¶^ (Table). During January 2018–September 2019, the national NPAFP rate was 15.9 per 100,000, ranging from 14.6 to 27.7 among provinces; the percentage of AFP cases with adequate stool specimens was 89% nationally, ranging from 86% to 92% among provinces.

**TABLE:** Acute flaccid paralysis (AFP) surveillance indicators and number of reported cases of wild poliovirus (WPV) and number and proportion of WPV-positive environmental surveillance samples, by region and period — Pakistan, January 2018–September 2019

Characteristic	Area							
	Pakistan total	Azad Jammu and Kashmir	Gilgit-Baltistan	Islamabad	Khyber Pakhtunkhwa	Punjab	Balochistan	Sindh
**2018 AFP surveillance indicators**								
No. of AFP cases	**12,276**	266	112	145	3,216	5,514	580	2,443
Nonpolio AFP rate*	**14.0**	12.8	17.1	21.2	20.6	12.2	14.2	12.7
% with adequate specimens^†^	**87**	88	85	80	86	88	87	88
**2019 AFP surveillance indicators**								
No. of AFP cases	**10,800**	273	115	140	2,400	5,207	465	2,200
Nonpolio AFP rate*	**16.2**	17.1	23.5	27.1	20	15.3	14.9	15
% with adequate specimens^†^	**88**	92	86	90	86	88	89	90
**Reported WPV cases**								
Jan–Jun 2018	**3**	—^§^	—	—	—	—	3	—
Jul–Dec 2018	**9**	—	—	—	8	—	—	1
Jan–Sep 2019	**72**	—	—	—	53	5	6	8
**Total Jan 2018–Sep 2019**	**84**	**0**	**0**	**0**	**61**	**5**	**9**	**9**
**Environmental surveillance no. of samples (%)**								
Jan–Jun 2018	**43 (13)**	NA	NA	2 (33)	13 (21)	6 (6)	6 (10)	16 (16)
Jul–Dec 2018	**96 (27)**	NA	NA	5 (83)	30 (39)	23 (23)	18 (30)	20 (19)
Jan–Sep 2019	**250 (43)**	NA	NA	3 (25)	31 (26)	63 (35)	45 (46)	108 (65)
**Total Jan 2018–Sep 2019**	**389**	**NA**	**NA**	**10**	**74**	**92**	**69**	**144**

**Abbreviation:** NA = not available.

* Per 100,000 children aged <15 years.

^†^ Adequate stool specimens are defined as two stool specimens of sufficient quality for laboratory analysis, collected ≥24 hours apart, both within 14 days of paralysis onset, and arriving in good condition at a World Health Organization–accredited laboratory with reverse cold chain maintained, without leakage or desiccation, and with proper documentation.

^§^ A dash indicates that no cases were reported in the area during the given period.

**Environmental surveillance.** Environmental surveillance supplements AFP surveillance through systematic sewage sampling (currently at 60 sites) and testing for poliovirus. During January 2018–September 2019, in addition to core reservoirs, poliovirus was detected continually from multiple nonreservoir sites, particularly those in Khyber Pakhtunkhwa (Bannu and South Waziristan), Punjab (Islamabad, Lahore, and Rawalpindi), and Sindh (Hyderabad and Sukkur) (Table). Among the same 51 sites sampled during January 2018–September 2019, 70 of 457 specimen (15%) were WPV1-positive in 2017, 74 of 459 (16%) in 2018 and 209 of 468 (45%) in 2019. Approximately 45% of all environmental sites tested positive in 2019, compared with 15% during the same period in 2018 and 16% in 2017.

## Epidemiology of WPV1 Cases

**FIGURE 1:**
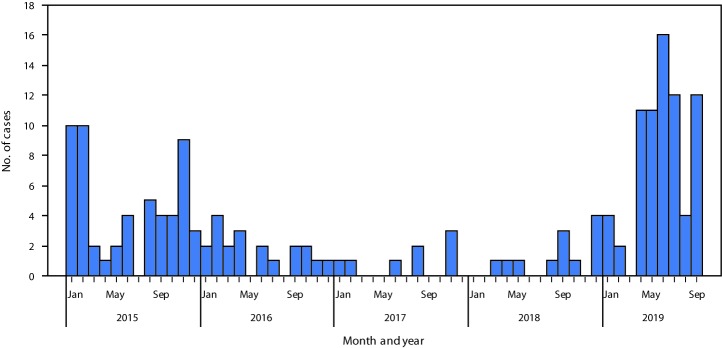
Cases of wild poliovirus type 1 by month — Pakistan, January 2015–September 2019

**FIGURE 2:**
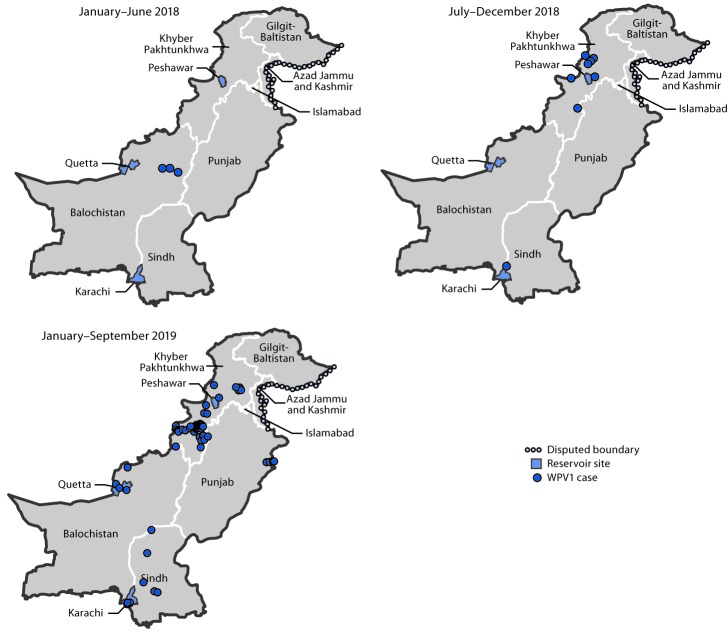
Location of reported wild poliovirus type 1 (WPV1) cases, by province and period — Pakistan, January 2018–September 2019

Twelve WPV1 cases were reported in Pakistan during 2018, a 50% increase from eight in 2017 (Figure 1). Seventy-two WPV1 cases have been reported during January–September 2019 among 22 districts in four provinces, compared with four during the same period in 2018 among four districts in two provinces. Of the 84 WPV1 cases with onset during January 2018–September 2019, 61 (73%) were from Khyber Pakhtunkhwa, nine (11%) from Balochistan, nine (11%) from Sindh, and five (6%) from Punjab (Table) (Figure 2). Among these 84 cases, ages of patients ranged from 2 to 144 months (median = 18 months). According to parental recall, nine (11%) patients had received zero OPV doses, 12 (14%) had received 1–3 doses, and 59 (70%) had received ≥4 doses. Four (5%) patients had unknown vaccination histories or are still being investigated. Among those who received ≥1 dose, two (2%) received only routine immunization and 49 (58%) only SIA doses.

Several viral genetic lineages persisted through the 2018–2019 low season (November–April) and, concomitant with the increase in the number of detected WPV1 cases, markedly expanded during 2019, particularly in Khyber Pakhtunkhwa. Among the five genetically distinct clusters (i.e., groups of polioviruses sharing ≥95% sequence identity in the viral capsid protein VP1) associated with AFP cases, during the reporting period, four were detected in Khyber Pakhtunkhwa.

## Discussion

Observations based on the geography of WPV1-positive environmental surveillance sites and viral genomic sequence diversity indicate that the Pakistan polio eradication program made substantial progress during 2015–2016 but, despite slight decreases in case numbers, progress stalled during 2017–2018 (*2*). The number of cases in 2019 to date has increased approximately fifteenfold from the same period in 2018, and the geographic distribution of WPV1-positive environmental surveillance specimens has expanded beyond the core reservoirs. The proportion of positive environmental surveillance specimens began to increase in mid-2017, heralding the subsequent increase in the number of paralytic cases in late 2018. The current status of polio eradication in Pakistan has serious global implications: the increased risk for WPV1 spreading beyond Pakistan’s borders is high; if transmission in Pakistan is not quickly controlled and back on track toward interruption, success of the Global Polio Eradication Initiative is threatened.

The Pakistan program’s failure in progress toward polio eradication is related to both community and program management challenges. Community challenges are increasingly strident refusals to vaccinate and children chronically missed by immunization activities. Because national and subnational SIAs have been occurring every 4–6 weeks, and there are frequent response campaigns after identification of polio cases and WPV1-positive environmental samples as well, campaign-fatigued communities are complaining that the government is not addressing other public health needs (e.g., nutrition and clean water) or other public services (*5*). The spread of false information, particularly through social media (e.g., that OPV contains pork products or causes sterility) has increased community resistance to vaccination (*6*). It is essential that the program counters false information, informs communities of the importance of vaccination, and engages and listens to communities to reestablish trust in the vaccination program. While starting to address these issues, the program has suspended SIAs in core reservoirs until December 2019. The Technical Advisory Group, an expert polio group comprising internal and external partners from a variety of backgrounds (e.g., virology, vaccines and vaccine delivery, epidemiology, and public health policy) that provides critical feedback on the polio program, recommended in August 2019 that SIAs subsequently be spaced ≥2 months apart to assist in community engagement (*7*). In addition, mobile populations are difficult to identify, track, and target for vaccination; however, the Pakistan program has enhanced internal efforts and is coordinating polio eradication activities with the Afghanistan program (i.e., vaccinations at border crossing, data sharing, and coordination of SIAs).

The Pakistan polio eradication program has grown complex in its management and operational organization. Management review in three districts of Karachi revealed overlapping terms of reference, delayed availability of information, systematic gaps in managerial oversight of decisions and activities, and an overall failure in staff members’ accountability when implementing SIAs (McKinsey and Company, unpublished report, 2019). The review concluded that meeting programmatic challenges might require managerial restructuring so that decision-making, oversight, and implementation occur as “One Team.” At the union council level, identifying the causes of operational failures in planning and supervision could enable the program to vaccinate those children who chronically have been missed. Local restructuring could improve oversight in such underperforming union councils, which has been considered an impediment to stopping WPV1 circulation in Karachi, Peshawar, and Quetta. Restructuring at national and provincial emergency operation centers and streamlining data flow could improve timely and effective decision-making.

The Pakistan polio eradication program has undertaken a series of management, communication, community engagement, and epidemiologic reviews that have identified essential gaps needing to be addressed. The national leadership has committed to implementing transformative changes, and maintaining this sense of urgency is essential. Managerial and operational weaknesses and gaps have been acknowledged, and the means to rectify them have been identified. Although responding to community concerns to minimize OPV refusal will take time, enhanced efforts (e.g., continual engagements with communities, countering false information, greater accountability, and more effective oversight) have already begun. The goal of interrupting WPV1 transmission in Pakistan is achievable but will require full and rapid implementation of Technical Advisory Group recommendations to improve program management and operational effectiveness.

**Summary**

**What is already known about this topic?**

Since 2016, Afghanistan and Pakistan have been the only countries reporting ongoing transmission of indigenous wild poliovirus type 1 (WPV1).

**What is added by this report?**

During January 2018–September 2019, the number of WPV1 cases in Pakistan increased, compared with the number during the previous 4 years. Sewage samples indicated wide WPV1 transmission, not only in the three major reservoir areas in three provinces, but also among other districts and provinces. Vaccine refusals, chronically missed children, community campaign fatigue, and poor vaccination management and implementation have exacerbated the situation.

**What are the implications for public health practice?**

Stopping WPV1 transmission will require continuing cross-border coordination with Afghanistan, gaining community trust, conducting high-quality campaigns, improving oversight of field activities, and improving managerial processes to unify eradication efforts.

## Footnotes

* Mass campaigns conducted for a brief period (days to weeks) in which 1 dose of oral poliovirus vaccine is administered to all children aged <5 years, regardless of vaccination history. Campaigns can be conducted nationally or subnationally (i.e., in portions of the country).

† Vaccination histories of children aged 6–23 months with acute flaccid paralysis who do not test positive for WPV are used to estimate OPV coverage of the overall target population and to corroborate national reported routine vaccination coverage estimates.

§ Lot quality assurance sampling is a rapid method used to assess the quality of vaccination activities after SIAs in predefined areas such as health districts (referred to as “lots”), using a small sample size. Lot quality assurance sampling involves dividing the population into lots and ascertaining receipt of vaccination by randomly selected persons within each lot. If the number of unvaccinated persons in the sample exceeds a predetermined value, then the lot is classified as having an unsatisfactory level of vaccination coverage, and mop-up activities are recommended.

¶ AFP surveillance quality is monitored by performance indicators that include 1) the detection rate of NPAFP cases and 2) the percentage of AFP cases with adequate stool specimens. WHO operational targets for countries with endemic poliovirus transmission are NPAFP detection rates of ≥2 cases per 100,000 population aged <15 years and adequate stool specimen collected from ≥80% of AFP cases. Adequate stool specimens are defined as two stool specimens of sufficient quality for laboratory analysis, collected ≥24 hours apart, both within 14 days of paralysis onset, and arriving in good condition at a World Health Organization–accredited laboratory with reverse cold chain maintained, without leakage or desiccation, and with proper documentation.
